# DMU-Net: A Dual-Stream Multi-Scale U-Net Network Using Multi-Dimensional Spatial Information for Urban Building Extraction

**DOI:** 10.3390/s23041991

**Published:** 2023-02-10

**Authors:** Peihang Li, Zhenhui Sun, Guangyao Duan, Dongchuan Wang, Qingyan Meng, Yunxiao Sun

**Affiliations:** 1 School of Geology and Geomatics, Tianjin Chengjian University, Tianjin 300384, China; 2 Key Laboratory of Soft Soil Engineering Character and Engineering Environment of Tianjin, Tianjin Chengjian University, Tianjin 300384, China; 3 Beijing Water Science and Technology Institute, Beijing 100048, China; 4 Aerospace Information Research Institute, Chinese Academy of Sciences, Beijing 100101, China; 5 University of Chinese Academy of Sciences, Beijing 100049, China; 6 Key Laboratory of Earth Observation of Hainan Province, Hainan Aerospace Information Research Institute, Sanya 572029, China

**Keywords:** GF-7 image, building extraction, nDSM, semantic segmentation, dual-stream network

## Abstract

Automatically extracting urban buildings from remote sensing images has essential application value, such as urban planning and management. Gaofen-7 (GF-7) provides multi-perspective and multispectral satellite images, which can obtain three-dimensional spatial information. Previous studies on building extraction often ignored information outside the red–green–blue (RGB) bands. To utilize the multi-dimensional spatial information of GF-7, we propose a dual-stream multi-scale network (DMU-Net) for urban building extraction. DMU-Net is based on U-Net, and the encoder is designed as the dual-stream CNN structure, which inputs RGB images, near-infrared (NIR), and normalized digital surface model (nDSM) fusion images, respectively. In addition, the improved FPN (IFPN) structure is integrated into the decoder. It enables DMU-Net to fuse different band features and multi-scale features of images effectively. This new method is tested with the study area within the Fourth Ring Road in Beijing, and the conclusions are as follows: (1) Our network achieves an overall accuracy (*OA*) of 96.16% and an intersection-over-union (*IoU*) of 84.49% for the GF-7 self-annotated building dataset, outperforms other state-of-the-art (SOTA) models. (2) Three-dimensional information significantly improved the accuracy of building extraction. Compared with RGB and RGB + NIR, the *IoU* increased by 7.61% and 3.19% after using nDSM data, respectively. (3) DMU-Net is superior to SMU-Net, DU-Net, and IEU-Net. The *IoU* is improved by 0.74%, 0.55%, and 1.65%, respectively, indicating the superiority of the dual-stream CNN structure and the IFPN structure.

## 1. Introduction

Buildings, as the basic geographical entity of cities, its spatial information are widely used in urban planning, disaster mitigation and prevention, population forecasting, and energy consumption [1,2,3,4]. With the rapid development of remote sensing technology, the spatial resolution of remote sensing images has reached the sub-meter level. The images have more spectral, texture, and spatial structure information, making it possible to refine and automatically extract buildings. Due to the density of urban buildings and the diversification of roof materials and structures, how to extract buildings accurately and quickly still face challenges.

In recent decades, there has been a great deal of research on building extraction. According to the type of data used, the methods of building extraction can be roughly divided into two categories: one is to extract buildings based on only a single data type, such as optical image, SAR image, LiDAR data, etc. [5,6,7]; The other is to extract buildings based on the fusion of multi-source data, such as optical image + LiDAR data fusion, optical image + SAR image fusion, etc. [8,9]. Compared to a single data type, the method of multi-source data fusion can obtain more features. For example, three-dimensional information can be obtained through LiDAR data, which helps to distinguish buildings and similar ground objects (such as roads, squares, etc.) [10]; Buildings with different heights, scales, and properties in SAR images have different scattering characteristics [11]. However, due to the high cost of LiDAR data acquisition and the interpretability of SAR images, it is still difficult to quickly and effectively obtain single large-scale buildings in cities [12,13]. At the same time, when the data of different sensors are fused, the registration error of different sensors and the change of ground objects caused by the gap in acquisition time have a certain impact on building extraction [14]. Very High Resolution (VHR) stereo image pairs can simultaneously acquire VHR multispectral images and digital surface model (DSM) data in a large area, which can effectively avoid the above errors and reduce the cost of LiDAR data acquisition. Gaofen-7 (GF-7), one of China’s most advanced stereo observation satellites, provides DSM data with an elevation root mean square error lower than 1 m [15]. Stereo images acquired by GF-7 have been used in three-dimensional information extraction and modeling of urban buildings, showing the power to describe the vertical structure of urban features [16,17]. However, there have been studies using DSM data constructed from Pleiades or WorldView stereo satellite imagery for building extraction [18,19]. The potential of GF-7 stereo pair in building extraction needs to be further verified.

There are two technical difficulties in building extraction. One is the regularity and integrity of building edge extraction, and the other is the huge difference in the size of different buildings and how to extract features about buildings of different scales efficiently. Based on the characteristics of GF-7, the fusion strategy of multimodal data should also be considered. Among them, nDSM data can enhance the detailed characteristics of buildings and improve the integrity of building segmentation, which can effectively solve the first problem. Two other problems, the multi-scale of buildings feature extraction and the fusion of multi-modal data, will be discussed in detail next.

Urban buildings are of various scales, and the sizes may vary dozens of times, so it is challenging for multi-scale building feature extraction. The traditional methods of extracting buildings rely on artificial design features, such as structure, texture, spectrum, and other features of buildings, which are classified by feature matching or machine learning [20,21,22,23,24,25]. However, the increase in intra-class variance and the decrease of inter-class variance in high-resolution remote sensing images make it more difficult to design features manually [26]. In recent years, with the development of deep learning, convolutional neural networks (CNNs) have been widely used in remote sensing image semantic segmentation. In particular, end-to-end networks, represented by fully convolutional neural networks, achieve pixel-level classification and become a new paradigm for semantic segmentation [27]. However, due to the repeated down-sampling of the network, the spatial relationship is lost, and the result of up-sampling is blurred, which is not sensitive to the boundaries and details of buildings. Later, the encoder-decoder network structure represented by the U-Net, up-sampling through deconvolution and introducing skip layer connections to fuse shallow features and deep features, retaining certain image details. However, U-Net has limited generalization ability, which is not conducive to extracting buildings of different scales [28]. One solution is to take images of different scales as input to obtain multi-scale features. Sun et al. input three image patches of different sizes into three different CNN models and finally sent the mixed features fused by the three models into the support vector machine to obtain complete building information, but the models were large, and the operation was complicated [29]. Another solution is to use the Spatial Pyramid Pooling (SPP) module to obtain multi-scale features by fusing feature maps of different sizes of receptive fields [30,31,32,33]. Based on the encoder-decoder structure, Deng et al. obtained multi-scale features by adding the atrous spatial pyramid pooling module at the end of the encoder [34]. However, the amount of calculation is large, as to adding the SPP module at the end of the encoder and ignoring the shallow characteristics of the model, and the size of different receptive fields needs to be determined by multiple experiments. Lin et al. proposed the feature pyramid structure (FPN) to construct semantic features at various scales through a hierarchical structure of lateral connections [35]. FPN is simple to operate, has a minimal amount of computation, is similar to the U-Net, and can be easily integrated into the U-Net backbone network. It has been used in related semantic segmentation tasks and achieved good results [36,37].

CNNs are mainly based on RGB images, which cannot be directly applied to multi-modal data, so the corresponding multi-modal fusion strategy is necessary. According to the different positions of fusion, it can be divided into three types: (1) Data-level fusion. Multi-modal data are fused before feature extraction using data superposition or dimensional reduction [38,39]. However, this strategy ignores the correlation between different modal data features. (2) Feature-level fusion. In the feature learning stage, the features of different modal data are fused [40,41]. This method fails to fully exploit the high-level features of individual modality data. (3) Decision-level fusion. The output results of different modal data are fused by averaging or voting [41,42]. This method fails to exploit individual modality data’s low-level and mid-level features fully. We propose a new fusion architecture with the U-Net as the basic network structure to fully use the low-level, mid-level, and high-level features of different modal data. The dual-stream CNN structure is used to extract the features of RGB images and NIR + nDSM images. The shallow and middle-level features of different modalities are fused with the deep features of the up-sampling stage with the help of the skip-layer connection structure, to avoid the loss of varying depth features.

To effectively extract the features of buildings of different scales and fully leverage both individual modal and cross-modal features, we proposed a simple and effective dual-stream multi-scale building extraction network named DMU-Net. The main contributions are as follows:

(1) The dual-stream structure of the DMU-Net can effectively extract the features of multi-modal data, and the building features of different scales can be effectively integrated through the IFPN structure.
(2) The fusion of three-dimensional data with two-dimensional data significantly improves the accuracy of urban building extraction.
(3) Compared with different semantic segmentation networks, the DMU-Net has higher accuracy while preserving edge details.

## 2. Study Area and Dataset

**Figure 1:**
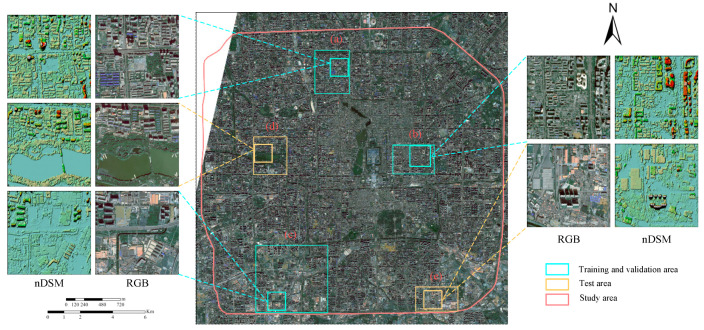
The study area is in Beijing. (**a**–**c**) as training and validation regions. (**d**,**e**) as test regions.

GF-7 was successfully launched in November 2019, and it is China’s first civilian sub-meter stereoscopic mapping satellite equipped with dual line scan cameras. GF-7 acquires 0.8 m front-view images (+26°) and 0.65 m rear-view images (−5°), and 2.6 m four-band multispectral images acquired by the rear-view multispectral camera. Taking the region within the Fourth Ring Road of Beijing as the study area, two adjacent GF-7 images obtained on 16 October 2020, were selected, with an area of about 302 km^2^, as shown in Figure 1. The study area covers major commercial and residential zones in Beijing, with dense buildings and diverse building structures.

To ensure the generalizability of the model, five distinct regions are selected from the study area, regions (a), (b), (c) as training and validation regions, and regions (d) and (e) as test regions. The ground-truth labels of the five regions are obtained by manual annotation. Regions (a), (b), and (c) cover the main types of buildings in the Fourth Ring Road, such as dense low-rise buildings, medium and high-rise buildings, and factory buildings. Region (d) contains other ground objects, such as water bodies, vegetation, squares, and roads, which can test the ability of different data to distinguish buildings from other ground objects. Region (e) contains large factory buildings, which can test the power of nDSM data and different network structures in extracting completeness and details of buildings. Based on the original image and ground-truth label, 640 pairs of image slices with a size of 512 × 512 pixels are randomly cropped from regions (a), (b), and (c) as the dataset, of which 540 pairs are selected as the training set, and the remaining 100 pairs are used as the validation set. Regions (d) and (e) are taken as test sets. The details of the dataset partition are shown in Table 1.

## 3. Materials and Methods

**Figure 2:**
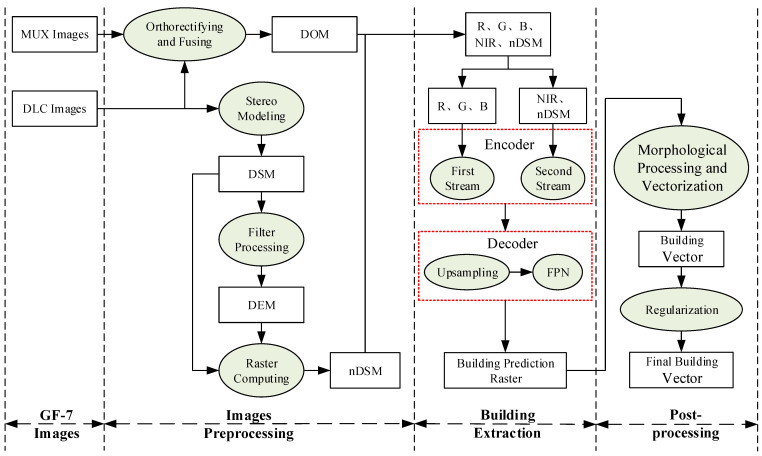
The flowchart of the proposed DMU-Net building extraction method.

The flowchart of the proposed method in this study is illustrated in Figure 2. It can be summarized by the following steps: (1) Data Preprocessing. The GF-7 backward multispectral image and backward panchromatic image are fused to obtain a VHR multispectral image, and the nDSM data is constructed based on the front and backward panchromatic images. (2) DMU-Net for urban building extraction. (3) Morphological operations and vector data regularization are used for post-processing.

### 3.1. Data Preprocessing

In this study, GF-7 image Sharpening and nDSM generation are performed by PCI Geomatica. Image Sharpening mainly includes ground control point (GCP) and tie point (TP) collection, orthorectification, and panchromatic sharpening. The 0.5 m resolution orthophoto from the Map World (TianDiTu) is used as the geographic reference image. The fast Fourier transform phase matching algorithm (FFTP) collects the GCPs between the rear-view panchromatic, multispectral image and the georeferenced image. GCPs with residual values greater than three are removed, and TPs are gathered to match panchromatic and multispectral images. Afterward, the points with larger errors are removed based on the residual report to complete the collection of GCPs and TPs. Finally, the backward panchromatic, multispectral images are orthorectified based on the rational functional model, and then the multi-resolution analysis algorithm is used for image sharpening. nDSM generation mainly includes the collection of GCPs and TPs, creating epipolar images, extracting DSM, and image filtering. The GCPs and TPs of the front-view panchromatic and forward panchromatic images are collected using FFTP. Then, the forward panchromatic image and the backward panchromatic image are determined as the left epipolar image and the right epipolar image to complete the creation of the epipolar images. The semi-global matching algorithm is used to generate DSM data [43]. Finally, a variety of filtering strategies are used to obtain digital elevation model (DEM) data, DEM data is subtracted from DSM data to obtain nDSM data, and the nDSM values of water bodies and buildings with missing height information are corrected by calculating the average value in the region or reassigning them.

### 3.2. DMU-Net Architecture

**Figure 3:**
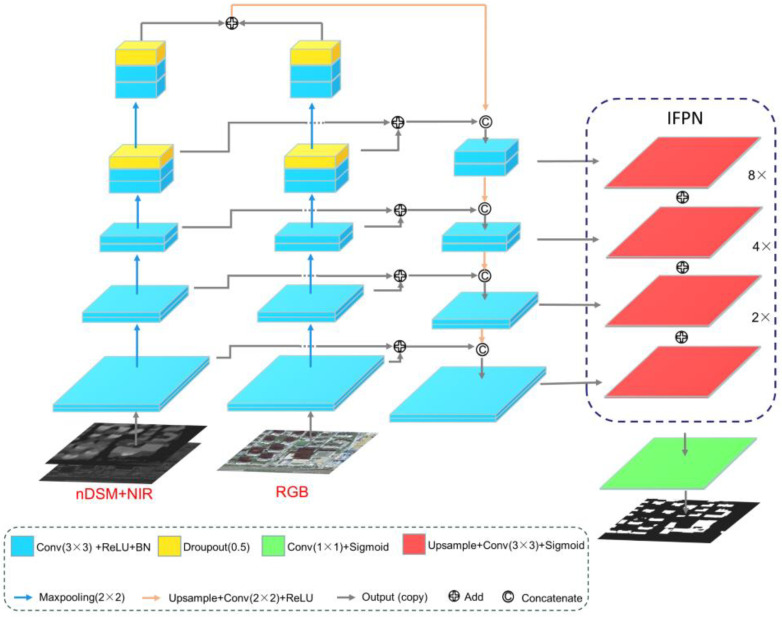
DMU-Net architecture.

We design DMU-Net that can fuse multi-modal data and multi-scale features to improve the accuracy of building extraction. As shown in Figure 3, inspired by previous studies [36,44,45,46], based on U-Net, we embed the two-stream CNN structure into the encoder of U-Net to obtain comprehensive features of different modal data. In the dual-stream structure, one stream is used to input the RGB image to get the feature of the two-dimensional spatial structure. The other stream is used to input the NIR + nDSM image, mainly used to obtain the feature of the three-dimensional spatial structure. In the decoder structure, it is still the up-sampling and skip connections of U-Net. Up-sampling is used to restore the dimension of the feature map, and the skip connection fuses the down-sampled feature map during the up-sampling process to realize the fusion of shallow and deep features, and to reduce the loss of original data details. The design of the independent dual-stream structure can extract multi-modal data features while avoiding the mutual interference between them and make full use of the image information of R, G, B, NIR, and nDSM bands [47]. Then, the IFPN structure is introduced in the decoding structure to fuse multi-scale information to account for the features of buildings of different scales. Finally, the sigmoid function is used to obtain the building segmentation map.

#### 3.2.1. Fusion Strategy

**Figure 4:**
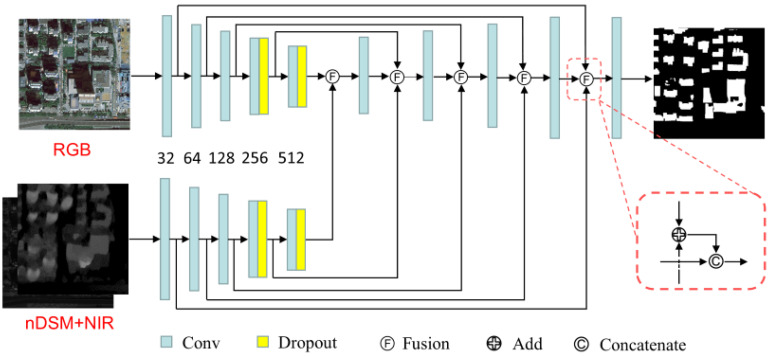
Dual-stream fusion structure.

As shown in Figure 4, the dual-stream architecture in the encoder has the same network structure and is independent of each other, and each stream performs four max-pooling down-sampling operations. Before each pooling, the features of the two streams are fused by the Add method and then fused with the corresponding up-sampled features in the decoder by the Concate process. To avoid a large number of parameters and memory consumption, the number of channels of the convolution kernel in the dual-stream structure is set to 32, 64, 128, 256, and 512 in turns. The up-sampling stage in the encoder adopts a method of first performing linear interpolation up-sampling, and then performing convolution. This method is equivalent to the transposed convolution operation, which is more effective than the simple interpolation up-sampling method and can effectively eliminate the aliasing effect. To speed up the training of the network, a BN layer is added after each 3 × 3 convolution for data normalization [48]. Dropout layers with a probability of 0.5 are added after the 4th and 5th groups of convolutional layers to enhance the robustness of the network and avoid overfitting [49].

#### 3.2.2. Improved Feature Pyramid Network

**Figure 5:**
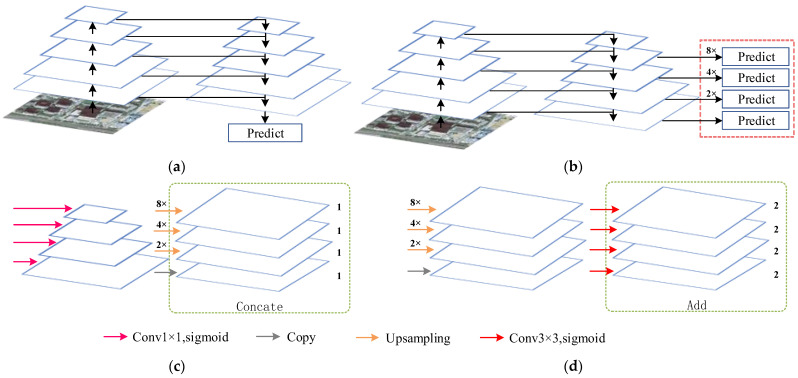
(**a**) U-Net, (**b**) FPN, (**c**) The FPN integration strategy proposed by Ji and Wei [45]; (**d**) IFPN.

FPN was first proposed to solve the multi-scale problem in object detection. The high-level features of low-resolution, high-semantic information and low-level features of high-resolution, low-semantic information are fused by top-to-bottom side connections. Therefore, the features of FPN at all scales have rich semantic information, which helps to extract objects at different scales. As shown in Figure 5a,b, U-Net and FPN have similar network structures. Ji et al. proposed a method to introduce the FPN module into the U-Net network (Figure 5c), and inspired by this, the IFPN is designed [45]. Different from the studies of Ji et al., in the decoder part, we first up-sampled the first three up-sampled feature graphs by 8×, 4×, and 2× linear interpolation to restore the original image size. Then 3 × 3 convolution is used to reduce the dimension of the feature map, as shown in Figure 5d. The 3 × 3 convolution has a larger receptive field than the 1 × 1 convolution, further enhancing the semantic features of buildings of different scales. Finally, the Add method is used to fuse the feature map.

### 3.3. Post-Processing of Buildings

#### 3.3.1. Digital Morphological Processing

The binary images of buildings predicted by CNN usually have noise and voids, which significantly interfere with the accuracy of building extraction and require a series of optimization processes. In this paper, the opening and closing operations in morphology are used to optimize the building prediction map, in which erosion and dilation are the basis of opening and closing operations [50]. The mathematical expressions for morphological erosion and dilation are:

(1) $$A\Theta B=\left\{z|{\left(B\right)}_{z}\subseteq A\right\}$$

(2) $$A⊕B=\left\{z|{\left(B\right)}_{z}\cap A\subseteq A\right\}$$

The corrosion of *B* to *A* is expressed as AΘB, the expansion of *B* to *A* is defined as A⊕B, *A* is a collection of building pixels, *B* is the structuring element, and *z* is the pixel value of the building.

The mathematical expressions for opening and closing operations are:

(3) A∘B=(AΘB)⊕B

(4) A•B=(A⊕B)ΘB

The open operation of *B* on *A* is expressed as A∘B, The closed operation of *B* on *A* is defined as A•B.

#### 3.3.2. Boundary Regularization

Although the building boundary after morphological processing has been smoothed, it cannot reflect the regular boundary of the buildings well. To better fit the boundary of the building, we adopt the polyline compression algorithm proposed by Gribov [51,52]. For building vector data, the algorithm’s goal is to find a point within the tolerance of all nodes of the vector line segment, so that the sum of the penalties of the synthetic polyline connected by all the points and the source polyline is the smallest. If there is a synthetic polyline with the same penalty, the polyline with the smallest square deviation from the source polyline is selected.

## 4. Experiments and Analysis

### 4.1. Evaluation Metric

To quantitatively evaluate the prediction results of the model, three evaluation metrics are calculated based on the confusion matrix: overall accuracy (*OA*), intersection-over-union (*IoU*), and *F*1-score (*F*1). The *OA* was used to assess the global accuracy of the extraction results, *IoU* was used to measure the overlap between building prediction results and real labels, and *F*1 took into account both the precision and recall of the model. The expression is as follows:

(5) $$OA=\frac{TP+TN}{TP+TN+FP+FN}$$

(6) $$IoU=\frac{precision\times recall}{precison+recall-precision\times recall}$$

(7) $$F1=\frac{2\times precision\times recall}{precision+recall}$$

(8) $$precision=\frac{TP}{TP+FP}$$

(9) $$recall=\frac{TP}{TP+FN}$$

where *TP* (true-positive) is the number of correctly identified building pixels, *FP* (false-positive) is the number of missed building pixels, *TN* (true-negative) is the number of correctly classified non-building pixels, and *FN* (false-negative) is the number of non-detected non-building pixels.

### 4.2. Experimental Details

**Figure 6:**
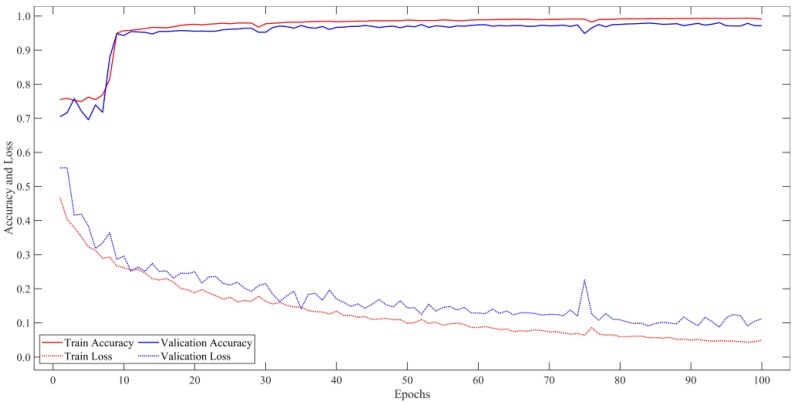
The Accuracy and Loss of DMU-Net for training the GF-7 self-annotated building dataset.

All experiments were performed on a desktop computer with 64-bit Windows 11. It is equipped with Intel (R) Core (TM) i5-11400 F CPU @ 2.60 GHz, a GPU of NVIDIA GeForce RTX 3060 with 12 GB RAM, and 16 GB memory (DDR4 3200 MHz). The methods in this paper are based on TensorFlow (version 2.5.0) and Keras (version 2.5.0), and the programming language is Python. The hyperparameters are set as follows: cross-entropy loss function and Adam optimization algorithm were used for 100 iterations of backward propagation, with four images in each batch, and the learning rate was 0.0001. In DMU-Net, RGB images are input in one stream, and NIR + nDSM images are input in the other stream. The changes in the accuracy and loss of the dataset with the number of training times are shown in Figure 6.

### 4.3. Results

#### 4.3.1. Comparative with SOTA Methods

To prove the excellent performance of DMU-Net, we use the GF-7 self-annotated building dataset, and select four excellent building extraction models for comparison, namely PSPNet, DeepLab v3+, EU-Net, and RU-Net [32,53,54,55]. Among them, the feature extraction network of PSPNet and DeepLab v3+ has the same structure as the single-stream CNN of SMU-Net.

**Figure 7:**
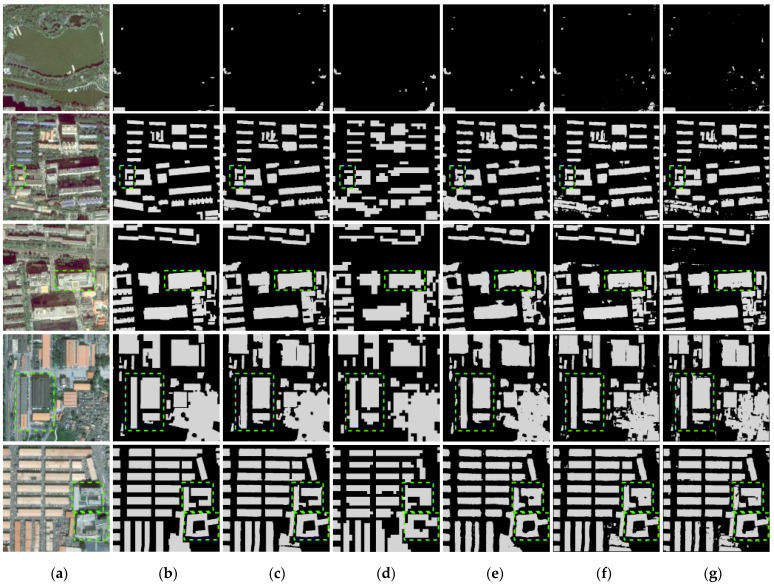
Building extraction results of different models. (**a**) Image (**b**) Label (**c**) DMU-Net (**d**) PSPNet (**e**) DeepLab V3+ (**f**) EU-Net (**g**) RU-Net.

As summarized in Table 2, which evaluated all three metrics on the GF-7 self-annotated building dataset, our proposed DMU-Net outperforms PSPNet, DeepLab v3+, EU-Net, and RU-Net and achieves a considerably high *IoU* (84.49%). Figure 7 shows the visual performances of the comparison results. As demonstrated in the Figure 7, DMU-Net shows the advantages of extracting complexly connected buildings marked in green in row 5. Other models’ building extraction results are missing or misclassifying non-building areas between buildings as buildings. For other green-marked buildings, DMU-Net can accurately extract buildings of different scales. At the same time, the results of DMU-Net are purer, while the results of EU-Net and RU-Net have white noise.

#### 4.3.2. Results of Building Extraction in the Study Area

We adopt DMU-Net to extract the buildings of the whole study area. To improve the binary results of DMU-Net, remove isolated points, and fill in holes, a mathematical morphology method is employed based on 3 × 3 rectangular structure elements; first, two open operations are performed, and then three closed operations are performed. Then further eliminate the small non-building noise to obtain the final building extraction result, and vectorize it based on the ArcGIS platform. To correct the deformation of the building’s vector boundary and better fit the building’s edge, the polyline compression algorithm is used to regularize the building vector data.

**Figure 8:**
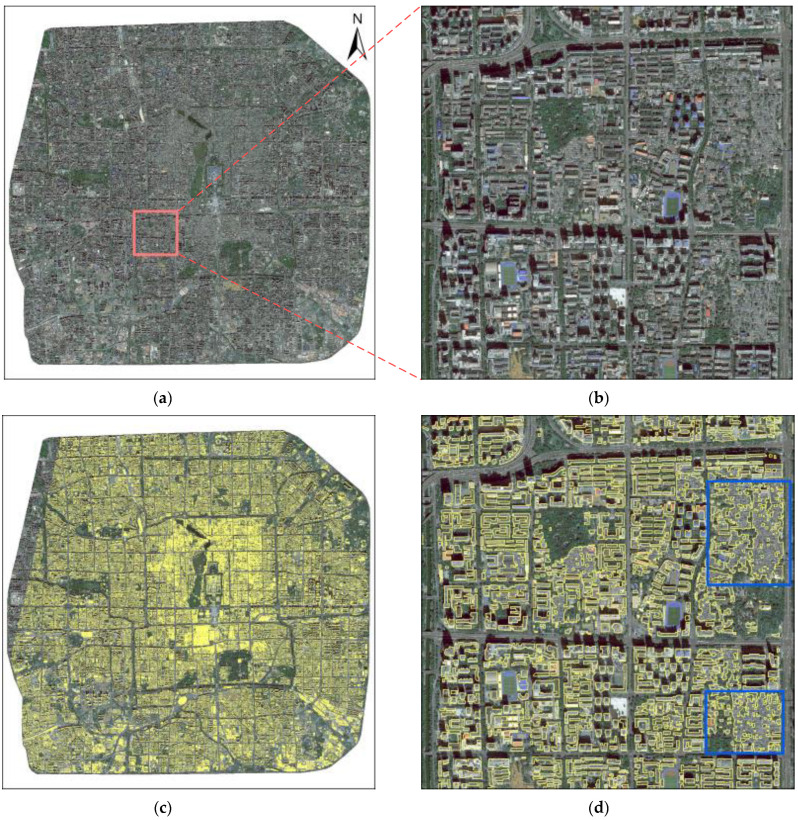
Extraction results of buildings in the study area. (**a**) the study area, (**b**) a typical local region, (**c**) building extraction result using DMU-Net, (**d**) local region result.

Figure 8c shows the vector result of buildings extracted by DMU-Net in the study area. Results showed that most of the buildings are correctly extracted. Due to the missing DSM data generated by GF-7, we have no extraction result of the building in the upper left area. To further analyze the results, we selected a typical local region from Figure 8b; this region contains high-rise independent buildings, contiguous low-rise buildings, and other ground objects similar to buildings. From Figure 8d, DMU-Net can completely extract large buildings with regular and complete edges. However, due to the low spatial resolution of the GF-7 image, the dense low-rise buildings (marked by the blue boxes) can only be extracted contiguously, and individual buildings cannot be distinguished.

## 5. Discussion

### 5.1. Comparative Analysis

#### 5.1.1. Validity of NIR and nDSM Data

**Figure 9:**
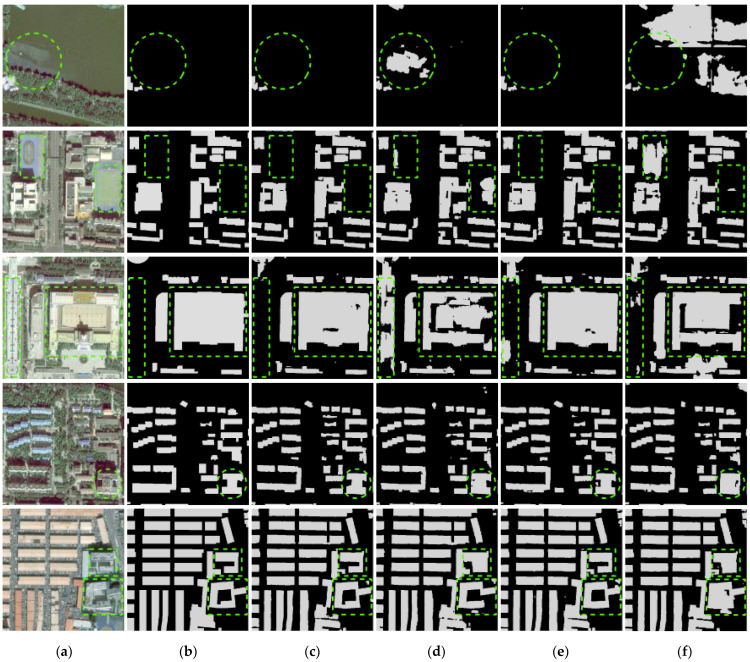
Building extraction results of models with different inputs. (**a**) Image (**b**) Label (**c**) M1: NIR + nDSM + RGB (**d**) M2: NIR + RGB (**e**) M3: nDSM + RGB (**f**) M4: RGB.

To explore the impact of NIR and nDSM data on building extraction, we fixed one of the streams to input RGB images, and the second stream input NIR, nDSM, and NIR + nDSM images, respectively. Based on the different inputs of the second stream, four different models, M1, M2, M3, and M4, are designed. Figure 9 shows the building extraction results of different models. Among them, M1 and M3 introduce nDSM images, which can effectively distinguish similar objects (such as playgrounds, squares, etc.), improve the integrity of large buildings extraction and avoid the adhesion of adjacent buildings. To further evaluate the effectiveness of our method, we quantitatively analyze the building extraction results of different models. As shown in Table 3, for the GF-7 self-annotated building dataset, M1 has the highest building extraction accuracy. Compared with M4, the *IoU* of M1, M2, and M3 have increased by 8.31%, 5.12%, and 7.61%, respectively, indicating that NIR and nDSM data help improve the accuracy of building extraction. At the same time, compared with M2, the *IoU* of M3 has increased by 2.49%; it shows that nDSM data contribute more to improving the accuracy of building extraction.

#### 5.1.2. Comparison of Different Network Structures

**Figure 10:**
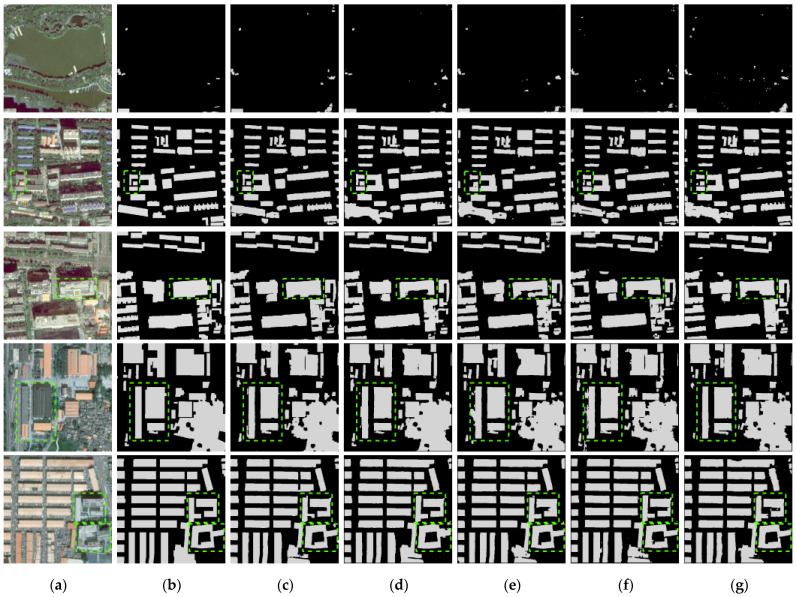
Building extraction results for different network structures. (**a**) Image; (**b**) Label; (**c**) DMU-Net; (**d**) DMU-Net (FPN); (**e**) SMU-Net; (**f**) DU-Net; (**g**) IEU-Net.

To verify the contribution of the dual-stream CNN structure and the IFPN structure to DMU-Net, the dual-stream with FPN model (DMU-Net (FPN)), the single-stream with IFPN model (SMU-Net), the dual-stream without IFPN model (DU-Net) and the single-stream without IFPN model (IEU-Net) were constructed for comparison (Table 4). For a fair comparison, the input data of all models contain RGB + NIR + nDSM images. Among them, one stream of DMU-Net, DMU-Net (FPN) and DU-Net input RGB images, and the other stream input NIR + nDSM images. SMU-Net and IEU-Net directly input RGB + NIR + nDSM images. As shown in Figure 10, DMU-Net has apparent advantages in the accurate extraction of adjacent buildings and the completeness of extensive buildings extraction. According to Table 5, using the dual-stream structure and the IFPN structure can effectively improve the accuracy of building extraction. DMU-Net compared with SMU-Net, DU-Net, and IEU-Net, the *IoU* of buildings increased by 0.74%, 0.55%, and 1.65%, respectively. In addition, DMU-Net compared with DMU-Net (FPN), the *IoU* of buildings increased by 0.22%, and the params and floating-point operations (FLOPs) of the model changed little, showing the advantages of IFPN. Compared with the single-stream CNN structure, the Trainable params and FLOPs of the two-stream CNN structure increase by about 1.6 times. IFPN structure had little effect on the model; the Trainable params and FLOPs did not increase by more than 0.02 M.

#### 5.1.3. Different Fusion Methods

Multimodal data fusion methods are divided into data-level fusion, feature-level fusion, and decision-level fusion. We used feature-level fusion. The building extraction accuracy of different fusion methods is shown in Table 6, the fusion method in this paper is the best in three indicators: *OA*, *IoU*, and *F*1. The *IoU* increased by 0.58% and 2.08%, respectively, compared with data-level fusion and decision-level fusion.

#### 5.1.4. Advantages of Regularization

**Figure 11:**
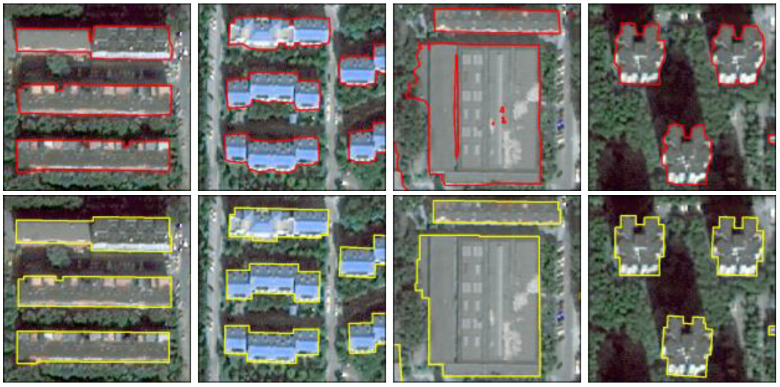
The vectorization result of building extraction. The first row is the vectorized result of the original prediction of the buildings; The second row is the results of the building after morphological processing and regularization of the original building prediction results.

To confirm that the regularization method adopted in this paper can effectively optimize the results of building extraction, we refer to the PoLiS metric proposed by Avbelj et al. [56]. We evaluate the similarity of all building vector predictions to the ground truth building vectors by computing the overall mean of PoLiS [57]. Smaller values indicate a higher similarity between the predicted building vectors and the actual building vectors. According to Table 7, although the three indicators of *OA*, *IoU*, and *F*1 of the building extraction results after regularization processing are slightly reduced, PoLiS is halved, indicating that the vector boundaries of buildings after regularization processing are more similar to actual buildings. Figure 11 shows that after morphological and regularization treatment, the edge of the building is more consistent with the natural shape of the building, and the holes in the building are eliminated.

### 5.2. Limitations and Future Works

Although the nDSM data constructed by GF-7 stereo pairs can significantly improve the accuracy of building extraction, compared with LiDAR data, the quality of nDSM data produced based on multi-view satellite images has shortcomings, such as insufficient precision of nDSM, and lack of height information of some buildings due to occlusion, which affects the performance of multi-view satellite data in building extraction. However, the main advantage of multi-view satellite imagery is that it can quickly obtain the height information of ground objects in a large area, and has strong timeliness and economic applicability. In the future, improving the quality of nDSM data generated from multi-view satellite data such as GF-7 is a promising exploration.

The DMU-Net performs the best effect on the GF-7 self-annotated building dataset. However, the two-stream structure has greater computational and memory overhead than the single-stream, which limits the applicability on different hardware. In the future, how to reduce the computational cost of the dual-stream CNN is a bottleneck. It must be overcome to ensure its wide application. More efficient multi-modal data fusion networks need to be proposed. In addition, the regularized post-processing method used in this paper needs to set complex calculation rules, which undoubtedly added to the task’s workload. It is an exciting direction to integrate the regularized method into the end-to-end segmentation model in the future.

## 6. Conclusions

In this paper, we propose a dual-stream multi-scale U-Net network named DMU-Net, to automatically and accurately extract urban buildings from GF-7 stereo images. For DMU-Net, the dual-stream CNN architecture is designed in the encoder to learn the multi-dimensional features of different modal data. The decoder introduces the IFPN structure to fuse features of different scales. Compared with four SOTA models, our model achieves the best results on the GF-7 self-annotated building dataset. In addition, the nDSM data constructed from GF-7 stereo images can help improve the accuracy of building extraction. In particular, it can help distinguish buildings from similar ground objects and improve the integrity of large buildings. In the future, we will develop more effective multimodal fusion models and regularization methods.

## Figures and Tables

**Table 1:** GF-7 Self-Annotated Building Dataset.

Region	Area (km^2^)	Buildings	Training Images	Validation Images	Test Images
(a)	6	1518	120	30	-
(b)	5	720	80	20	-
(c)	18	2328	340	50	-
(d)	5	629	-	-	49
(e)	4	436	-	-	40
Sum	38	5631	540	100	89

**Table 2:** Building extraction accuracy corresponding to different network structures.

Model	*OA* (%)	*IoU* (%)	*F*1 (%)
DMU-Net	96.16	84.49	91.59
PSPNet [53]	92.25	71.06	83.08
DeepLab V3+ [54]	95.29	81.21	89.63
EU-Net [32]	95.64	82.41	90.36
RU-Net [55]	94.89	79.71	88.71

**Table 3:** Accuracy of building extraction based on different modal data combinations.

Model	First Stream	Second Stream	*OA* (%)	*IoU* (%)	*F*1-Score (%)
M1	NIR + nDSM	RGB	96.16	84.49	91.59
M2	NIR	RGB	95.21	81.30	89.69
M3	nDSM	RGB	95.91	83.79	91.18
M4	-	RGB	93.56	76.18	86.48

**Table 4:** Models of different network structures.

Model	States
DMU-Net	The method proposed in this paper.
DMU-Net (FPN)	The data is input by a dual-stream CNN and the FPN structure is retained.
SMU-Net	The data is input by a single-stream CNN and the IFPN structure is retained.
DU-Net	The IFPN structure is removed and the dual-stream CNN structure is retained.
IEU-Net	Data are input by single-stream CNN and the IFPN structure is removed [46].

**Table 5:** The accuracy of building extraction of different network structures.

Model	*OA* (%)	*IoU* (%)	*F*1 (%)	Trainable Params (M)	FLOPs (M)
DMU-Net	96.16	84.49	91.59	12.49	24.97
DMU-Net (FPN)	96.08	84.27	91.47	12.48	24.95
SMU-Net	96.00	83.75	91.15	7.78	15.54
DU-Net	96.08	83.94	91.27	12.48	24.95
IEU-Net	95.75	82.84	90.61	7.77	15.53

**Table 6:** Building extraction accuracy of different fusion methods.

Model	*OA* (%)	*IoU* (%)	*F*1 (%)
data-level fusion	95.96	83.69	91.12
feature-level fusion (ours)	96.12	84.27	91.46
decision-level fusion	95.65	82.19	90.22

Note: Due to memory limitations, each batch takes three training images.

**Table 7:** Accuracy of building prediction vector results.

Method	*OA* (%)	*IoU* (%)	*F*1 (%)	PoLiS
the results of original prediction	96.16	84.49	91.59	16.53
the results after morphological processing and regularization	95.95	83.73	91.15	8.48

## Data Availability

The data are not publicly available due to [privacy restrictions].
